# Diabetes does not attenuate the benefit of physiology-guided complete revascularization in older patients with myocardial infarction

**DOI:** 10.1186/s12933-026-03165-5

**Published:** 2026-04-14

**Authors:** Alberto Sarti, Filippo Maria Verardi, Caterina Cavazza, Andrea Erriquez, Gianni Casella, Vincenzo Guiducci, Raul Moreno, Javier Escaned, Federico Marchini, Marta Cocco, Serena Caglioni, Jacopo Farina, Giuseppe Vadalà, Alessandro Capecchi, Francesco Gallo, Enrico Cerrato, Alberto Menozzi, Jose Luiz Diez Gil, Ignacio Amat Santos, Marco Ruozzi, Marco Barbierato, Andrea Picchi, Rita Pavasini, Roberto Scarsini, Matteo Tebaldi, Gianluca Campo, Simone Biscaglia

**Affiliations:** 1 Cardiology Unit, Azienda Ospedaliero Universitaria di Ferrara, Via Aldo Moro 8, 44124 Ferrara, Italy; 2 Cardiovascular Department, Infermi Hospital, Viale Luigi Settembrini 2, 47923 Rimini, Italy; 3 Cardiology Unit, Ospedale Maggiore, Largo Nigrisoli 2, 40133 Bologna, Italy; 4 Cardiology Unit, Azienda USL-IRCCS Reggio Emilia, S. Maria Nuova Hospital, Viale Risorgimento 80, 42123 Reggio Emilia, Italy; 5 Centro de Investigación Biomédica en Red en Enfermedades Cardiovasculares (CIBERCV), Madrid, Spain; 6 Instituto de Investigación Hospital La Paz (IDIPAZ), University Hospital La Paz, Madrid, Spain; 7 Hospital Clínico San Carlos IDISCC, Complutense University of Madrid, Calle del Prof Martin Lagos s/n, 28040 Madrid, Spain; 8 Azienda Ospedaliero Universitaria Policlinico Paolo Giaccone, Palermo, Italy; 9 UOC Cardiologia, Ospedale Dell’Angelo, Mestre, Venice, Italy; 10 Interventional Cardiology Unit, San Luigi Gonzaga University Hospital, Orbassano and ASLTO3 Infermi Hospital, Rivoli, Turin, Italy; 11 Cardiology Unit, S. Andrea Hospital, ASL5 Liguria, La Spezia, Italy; 12 Centro de Investigation Biomedica en Red en Enfermedades Cardiovasculares, Cardiology Department, H. Universitario y Politécnico La Fe, Valencia, Spain; 13 Cardiology Department, Centro de Investigación Biomédica en Red en Enfermedades Cardiovasculares (CIBERCV), Hospital Clínico Universitario, Valladolid, Spain; 14 Cardiology Unit, Ospedale Civile di Baggiovara, Baggiovara, Modena Italy; 15 Cardiovascular Department, Azienda Unità Sanitaria Locale (AUSL) Toscana Sud-Est, Misericordia Hospital, Grosseto, Italy; 16 Azienda Ospedaliero Universitaria Integrata di Verona, Verona, Italy; 17 Cardiology Unit, Ospedale degli Infermi, ASL Romagna, Faenza, RA Italy; 18 Cardiology Unit, Azienda Ospedaliera Universitaria S. Anna, Via Aldo Moro 8, 44124 Cona, FE Italy

**Keywords:** Myocardial infarction, Multivessel disease, Diabetes, Percutaneous coronary intervention, Revascularization

## Abstract

**Background:**

In patients with myocardial infarction (MI) and multivessel disease, diabetes mellitus is associated with more diffuse coronary atherosclerosis and worse clinical outcomes, often influencing revascularization decisions. The Functional Assessment in Elderly MI Patients with Multivessel Disease (FIRE) trial demonstrated the superiority of physiology-guided complete revascularization in older patients with MI. Whether this benefit is preserved in patients with diabetes remains uncertain.

**Methods:**

In FIRE, 1445 patients aged ≥ 75 years with MI and multivessel disease were randomized to culprit-only or physiology-guided complete revascularization. In this prespecified analysis, outcomes were assessed according to diabetes status. The primary endpoint was a composite of death, MI, stroke, or revascularization at 3 years. The key secondary endpoint was cardiovascular death or MI. The safety endpoint included contrast-associated acute kidney injury, stroke, or Bleeding Academic Research Consortium type 3–5 bleeding.

**Results:**

Among 1445 patients, 463 (32%) had diabetes. After adjustment for baseline characteristics, diabetes was independently associated with a higher risk of the primary endpoint (hazard ratio [HR] 1.26, 95% confidence interval [CI] 1.02–1.56) and heart failure (HR 1.35, 95% CI 1.01–1.83) at 3 years. Physiology-guided complete revascularization reduced the primary outcome in both patients with diabetes (HR 0.70, 95% CI 0.50–0.97) and without diabetes (HR 0.75, 95% CI 0.58–0.97), with no evidence of effect modification by diabetes status (p for interaction = 0.712). Similar consistency was observed for the key secondary and safety endpoints.

**Conclusions:**

In older patients with MI and multivessel disease, physiology-guided complete revascularization reduces ischemic events irrespective of diabetes status, supporting its use in elderly diabetic patients.

*Trial registration* ClinicalTrials.gov Identifier NCT03772743.

**What is currently known about this topic?**

Diabetes worsens coronary disease; coronary artery bypass grafting favored in stable cases; little data in elderly with acute coronary syndrome.

**What is the key research question?**

Does diabetes modify the benefit of physiology-guided complete revascularization in elderly patients with myocardial infarction?

**What is new?**

Complete revascularization reduces events in diabetics and non-diabetics with no safety difference.

**How might this study influence clinical practice?**

Diabetes should not discourage complete revascularization in elderly acute coronary syndrome patients.

## Background

The global prevalence of diabetes is steadily increasing, driven by population ageing and a growing burden of metabolic disease in the elderly [1]. Among patients presenting with acute coronary syndromes (ACS), approximately one quarter have established diabetes, and up to half may have undiagnosed diabetes or prediabetes at the time of hospitalization [2−4]. In this context, diabetes is associated with a two- to three-fold higher risk of mortality and a greater prevalence of multivessel coronary artery disease (CAD) compared with nondiabetic patients [5−9]. Despite the high-risk profile of these patients, evidence guiding revascularization strategies in diabetes largely derives from clinical settings that differ substantially from contemporary elderly ACS populations. The FREEDOM trial enrolled relatively young patients with diabetes and stable multivessel CAD, excluded ACS, and demonstrated superior outcomes with coronary artery bypass grafting compared with percutaneous coronary intervention (PCI) [10]. Conversely, the COMPLETE trial focused on younger patients with ST-segment elevation myocardial infarction (STEMI), addressing a distinct clinical scenario [11]. Although a prespecified subanalysis of COMPLETE suggested that complete revascularization was effective irrespective of diabetes status, these findings were confined to younger STEMI patients [12]. Whether these results can be extrapolated to older patients presenting with both STEMI and non-ST-segment elevation MI (NSTEMI)—who are frequently unsuitable for surgical revascularization and often perceived as candidates for a more conservative percutaneous approach—remains uncertain. In clinical practice, diabetes may still influence decision-making toward a more cautious revascularization strategy in elderly patients with multivessel disease. The present prespecified subanalysis of the FIRE trial was therefore designed to determine whether the clinical benefit of physiology-guided complete percutaneous revascularization is preserved in diabetic patients within a uniquely high-risk population: older patients with myocardial infarction and multivessel coronary artery disease.

## Methods

This was a prespecified subgroup analysis of the FIRE trial. The FIRE trial was conceived as a multicenter, investigator-initiated, randomized, superiority trial comparing the efficacy of physiology-guided complete revascularization versus a culprit-only strategy in older MI patients with multivessel disease. The design, baseline characteristics, and primary results of the trial have been detailed in previous publications [10, 14, 15]. All enrolled patients provided written informed consent, and the trial protocol was approved by the institutional review board at each participating center. The trial was registered on ClinicalTrials.gov with the identifier NCT03772743. The corresponding author had full access to all the trial data and takes responsibility for its integrity and the data analysis. The data underlying this article are available on reasonable request to the FIRE study Executive Committee. Follow-up events were collected through scheduled clinical visits according to the study protocol, as previously described in the main FIRE trial reports [13, 14].

### Study patients

Patients were eligible if they had the following inclusion criteria: (i) 75 years or older, (ii) hospital admission with either STEMI or NSTEMI, (iii) successful PCI of the culprit lesion, and (iv) at least one non-culprit coronary artery lesion with a minimum diameter of 2.5 mm and a diameter stenosis of 50–99% [10, 15, 16] Exclusion criteria were the inability to distinctly identify a culprit lesion, the presence of the non-culprit lesion in the left main, planned, or prior surgical revascularization, and a life expectancy of less than one year [10, 15, 16]. A non-culprit lesion classified as chronic total occlusion was not eligible as target non-culprit lesion for the study.

### Study procedures

Patients were randomized between July 18, 2019, and October 25, 2021 [10, 15, 16]. Patients who had been randomly assigned to physiology-guided complete revascularization received physiological assessment of non-culprit lesions using wire-based (hyperemic or non-hyperemic) and/or angiography-based (quantitative flow ratio, Medis Medical Imaging Systems B.V, Leiden, The Netherlands) measurements [10, 14, 15]. All non-culprit lesions deemed functionally significant were subjected to PCI with subsequent stent implantation [10, 15, 16]. Conversely, patients assigned to culprit-only revascularization did not receive revascularization for non-culprit lesions [10, 15, 16]. In both treatment groups, the implantation of sirolimus-eluting biodegradable-polymer ultrathin stents (Supraflex Cruz, Sahajanand Medical Technologies Ltd.) was strongly recommended. All individuals within both treatment arms received optimal medical therapy in accordance with established guidelines.

### Study endpoints

The primary outcome was a composite endpoint of death, MI, stroke, or ischemia-driven coronary revascularization occurring within three years of randomization. The key secondary outcome was the three-year composite endpoint of cardiovascular death or MI. Other secondary outcomes comprised the individual components of the composite endpoints, stent thrombosis and heart failure. The safety outcome was a composite of contrast-associated acute kidney injury (CA-AKI), stroke, or bleeding defined as type 3, 4, or 5 by the Bleeding Academic Research Consortium (BARC) at three years [17, 18]. Outcome events were adjudicated according to definitions of the Academic Research Consortium (ARC) and BARC consensus documents [17, 18]. All events were reported by investigators and analyzed and adjudicated by an independent clinical evaluation committee, who was blinded to the randomization arm.

### Statistical analysis

In the present analysis, patients were divided according to having or not at diabetes mellitus and their assigned randomization arm. Diabetes mellitus was defined as a documented history of diabetes and/or treatment with glucose-lowering medications at baseline. Statistical analysis was conducted in accordance with the intention-to-treat principle, where all patients were assessed based on their designated treatment group. The normal distribution of continuous variables was assessed through the Shapiro-Wilk test. Continuous variables were summarized with means (SD) or median [IQR], and comparisons were executed using the Student t-test or the Wilcoxon test, as appropriate. Categorical variables were presented as frequencies and percentages, and comparative analyses were conducted utilizing either the Pearson Chi-square or Fisher exact test, in alignment with appropriateness. Time-to-event data were evaluated with the use of Kaplan–Meier estimates and Cox proportional-hazards models, dividing the study population according to diabetes status and/or randomization arm. In case of imbalance in baseline characteristics within subgroups, analyses were adjusted for potential confounders. The proportionality assumption was tested using Schoenfeld residuals and was met (*p* > 0.05 for all outcomes). Estimates and confidence intervals for the outcomes that included cardiovascular death were adjusted for the competing risk of non-cardiovascular death. Other secondary and safety outcomes were adjusted for the competing risk of death. Subsequently, we conducted a Cox regression analysis with interaction testing to determine whether the effect of revascularization strategy on the prespecified endpoints was consistent across patients with or without diabetes mellitus. The interaction test was carried out using likelihood ratio tests of the null hypothesis that the interaction coefficient was zero. The statistical analyses were performed using the R statistical software (Foundation for Statistical Computing, Vienna, Austria).

## Results

The FIRE trial enrolled 1445 patients with MI across Italy, Spain, and Poland. Of these, 463 patients (32%) had diabetes mellitus (culprit-only arm *n* = 233, complete arm *n* = 230). The remaining 982 patients (68%) did not have diabetes mellitus (culprit-only arm *n* = 492, complete arm *n* = 490). Among the 463 patients with diabetes included in the present analysis, 127 were treated with insulin, whereas 336 were not receiving insulin therapy. Significant differences in baseline characteristics were observed between the two subgroups (Table 1). At baseline, patients with diabetes mellitus were slightly younger, had a higher prevalence of hypertension, lower estimated glomerular filtration rate at admission, a greater history of prior percutaneous coronary intervention, and more frequently presented with non–ST-segment–elevation myocardial infarction compared with those without diabetes mellitus (Table 1). Across randomization arms (physiology-guided complete revascularization vs. culprit-only revascularization), baseline medical history and clinical presentation were well balanced within both the diabetes and non-diabetes subgroups (Table 1). Angiographic characteristics of non-culprit lesions are reported in Table 2, including the lesion SYNTAX score. In the FIRE trial, anatomical complexity was assessed at the lesion level, consistent with the physiology-guided design of the study.

**Table 1:** Baseline characteristics according to the presence of diabetes mellitus and randomization arm

Characteristic				No DM		DM	
	No DM (*n* = 982)	DM (*n* = 463)	*p*	Culprit only (*n* = 492)	Complete (*n* = 490)	Culprit only (*n* = 233)	Complete (*n* = 230)
Age – years	81.2 ± 4.6	80.3 ± 4.1	< 0.001	81.2 ± 4.8	81.3 ± 4.5	80.3 (4.2)	80.4 (3.9)
Female sex – no. (%)	617(62.8)	300 (64.8)	0.47	307 (62.4)	310 (63.3)	153 (65.7)	147 (63.9)
Medical history – no. (%)							
Hypertension	770 (78.4)	415 (89.6)	< 0.001	387 (78.7)	383 (78.2)	205 (88)	210 (91.3)
Dyslipidemia	495 (50.4)	264 (57)	0.019	246 (50)	249 (50.8)	129 (55.4)	135 (58.7)
Current smoker	90 (9.2)	33 (7.1)	0.20	43 (8.7)	47 (9.6)	19 (8.2)	14 (6.1)
Prior MI	133 (13.5)	87 (18.8)	0.01	67 (13.6)	66 (13.5)	49 (21)	38 (16.5)
Prior PCI	148 (15.1)	109 (23.5)	< 0.001	81 (16.5)	67 (13.7)	55 (23.6)	54 (23.5)
History of AF	138 (14.1)	62 (13.4)	0.73	75 (15.2)	63 (12.9)	34 (14.6)	28 (12.2)
eGFR < 60 ml/min	420 (42.8)	243 (52.5)	< 0.001	213 (43.3)	207 (42.2)	120 (51.5)	123 (53.5)
PAD	148 (15.1)	101 (21.8)	0.002	77 (15.7)	71 (14.5)	50 (21.5)	51 (22.2)
CVA	89 (9.1)	30 (6.5)	0.095	51 (10.4)	38 (7.8)	12 (5.2)	18 (7.8)
Clinical presentation							
NSTEMI	598 (60.9)	338 (73.0)	< 0.001	301 (61.2)	297 (60.6)	168 (72.1)	170 (73.9)
STEMI	384 (39.1)	125 (27.0)		191 (38.8)	193 (39.4)	65 (27.9)	60 (26.1)
Killip ≥ 2 – no (%)	262 (26.7)	150 (32.4)	0.025	137 (27.8)	125 (25.5)	71 (30.5)	79 (34.3)
LVEF – %	49.6 (10.6)	48.4 (11.0)	0.061	49.3 (10.8)	49.9 (10.4)	48.5 (11.2)	48.3 (10.8)
Radial access – no (%)	930 (94.7)	426 (92.0)	0.091	458 (93.1)	472 (96.3)	214 (91.8)	212 (92.2)
Culprit vessel – no. (%)							
Left main coronary artery	47 (4.8)	29 (6.3)	0.68	26 (5.3)	21 (4.3)	15 (6.4)	14 (6.1)
Left anterior descending artery	450 (45.8)	209 (45.1)		224 (45.5)	226 (46.1)	106 (45.5)	103 (44.8)
Circumflex artery	187 (19.0)	82 (17.7)		97 (19.7)	90 (18.4)	36 (15.5)	46 (20)
Right coronary artery	281 (28.6)	132 (28.5)		138 (28)	143 (29.2)	71 (30.5)	61 (26.5)
Ramus Intermedius artery	17 (1.7)	11 (2.4)		7 (1.4)	10 (2)	5 (2.1)	6 (2.6)
N. of stents	1.5 ± 0.8	1.5 ± 0.9	0.44	1.4 ± 0.7	1.4 ± 0.8	1.5 ± 0.7	1.5 ± 0.9
Stent length (mm)	36 ± 22	38 ± 24	0.17	36 ± 22	36 ± 22	38.5 ± 24	37.7 ± 24

*MI* myocardial infarction,* PCI* percutaneous coronary intervention,* AF* atrial fibrillation,* eGFR* glomerular filtration rate, calculated by EPI-CKD formula,* PAD* peripheral artery disease,* CVA* cerebrovascular accident,* STEMI* ST-segment elevated myocardial infarction,* NSTEMI* non-ST-segment elevation myocardial infarction,* LVEF* left ventricular ejection fraction

**Table 2:** Baseline characteristics of non-culprit vessels

Characteristic				No DM		DM	
	No DM	DM	*p*	Culprit only	Complete	Culprit only	Complete
Patients	982	463		492	490	233	230
Non-culprit vessel – no. (%)	1291	605		642	649	308	297
Left anterior descending artery	402 (31.1)	185 (30.6)	0.67	201 (31.3)	201 (31)	90 (29.2)	95 (32)
Circumflex artery	422 (32.7)	204 (33.7)		211 (32.9)	211 (32.5)	107 (34.7)	97 (32.7)
Right coronary artery	427 (33.1)	203 (33.6)		216 (33.6)	211 (32.5)	104 (33.8)	99 (33.3)
Ramus Intermedius artery	40 (3.1)	13 (2.1)		14 (2.2)	26 (4)	7 (2.3)	6 (2)
Quantitative coronary analysis							
RVD (mm)	2.67 ± 3	2.76 ± 3.3	0.48	2.7 ± 2	2.6 ± 1	2.7 ± 3.2	2.9 ± 4.9
%DS (%)	60.5 ± 15	60.8 ± 29	0.71	59.5 ± 16.6	61.5 ± 15	58.9 ± 16	63 ± 38
Lesion length (mm)	16 ± 11	15.7 ± 10	0.65	15.4 ± 11.1	16.6 ± 11.8	14.3 ± 9.5	17.2 ± 11
ACC/AHA B2/C, no. (%)	531 (41,2)	258 (42.7)	0.95	273 (42.6)	258 (39.8)	116 (37.7)	142 (47.8)
Lesion SYNTAX score, no.	4.27 ± 3	4.38 ± 3	0.51	4.2 ± 2.9	4.4 ± 3.2	4.2 ± 2.8	4.6 ± 3.7
Treatment – no. (%)							
Positive physiology	292 (22.6)	138 (22.8)	0.93		292 (45)		138 (46.5)
PCI	292 (22.6)	138 (22.8)	0.93		292 (45)		138 (46.5)

%DS indicates percentage diameter stenosis; *ACC* American College of Cardiology, *AHA* American Heart Association, *PCI* percutaneous coronary intervention, *RVD* reference vessel diameter; and *SYNTAX* Synergy Between PCI With TAXUS and Cardiac Surgery

### Clinical outcomes of diabetes versus non-diabetes subgroups

The occurrence of the adverse events stratified according to subgroups is reported in Table 3. At univariate analysis, patients with diabetes were at higher risk for the primary endpoint [144 (31.1%) vs. 237 (24.1%)] and death at three years [99 (19.2%) vs. 159 (16.2%)] (Table 3). After correction for potential confounding factors (age, hypertension, sex, previous MI, eGFR, peripheral artery disease, Killip class ≥ 2, localization of the culprit vessel, clinical presentation), patients with diabetes remained at higher risk of the primary endpoint at 3-year (HR 1.26, 95%CI 1.02–1.56) and Heart Failure at 3-year [101 (21.8%) vs. 145 (14.8%); (HR 1.35, 95%CI 1.01–1.83)] (Table 3). The occurrence of stent thrombosis did not differ between subgroups (Table 3). Regarding safety endpoint, no significant differences were observed for any of the analyzed outcomes (Table 3).

**Table 3:** Clinical outcomes according to subgroups (diabetes vs. no-diabetes)

Outcome		No diabetes (*n* = 982)		Diabetes (*n* = 463)	*p*	Adjusted HR (95%CI) *	*p*
Primary outcome							
Composite of death, myocardial infarction, stroke, or ischemia-driven revascularization	no. (%)	237 (24.1)		144 (31.1)	0.003	1.26 (1.02–1.56)	0.036
	HR (95%CI)	1.37 (1.11–1.68)					
Secondary outcomes							
Cardiovascular death, myocardial infarction	no. (%)	135 (13.7)		89 (19.2)	0.058	1.27 (0.94–1.7)	0.116
	HR (95%CI)	1.31 (0.99–1.74)					
Death	no. (%)	159 (16.2)		99 (21.4)	0.015	1.28 (0.98–1.66)	0.066
	HR (95%CI)	1.37 (1.06–1.76)					
Cardiovascular death	no. (%)	84 (8.6)		49 (10.6)	0.223	1.26 (0.87–1.82)	0.221
	HR (95%CI)	1.24 (0.88–1.77)					
Myocardial infarction	no. (%)	58 (5.9)		54 (11.7)	0.173	1.28 (0.79–2.06)	0.322
	HR (95%CI)	1.38 (0.87–2.21)					
Stroke	no. (%)	22 (2.2)		10 (2.2)	0.775	1.09 (0.45–2.67)	0.847
	HR (95%CI)	1.13 (0.48–2.67)					
Ischemia-driven coronary revascularization	no. (%)	67 (6.8)		45 (9.7)	0.641	1.09 (0.69–1.73)	0.708
	HR (95%CI)	1.11 (0.71–1.74)					
Heart failure	no. (%)	145 (14.8)	101 (21.8)		0.087	1.35 (1.01–1.83)	0.049
	HR (95%CI)	1.29 (0.96–1.73)					
Stent thrombosis	no. (%)	9 (0.9)	8 (1.7)		0.847	1.11 (0.21–5.85)	0.898
	HR (95%CI)	0.85 (0.16–4.39)					
Safety outcomes							
Composite of CA-AKI, stroke, BARC type 3–5 bleeding	no. (%)	225 (22.9)	131 (28.3)		0.237	1.12 (0.86–1.47)	0.396
	HR (95%CI)	1.17 (0.90–1.51)					
CA-AKI	no. (%)	150 (15.3)	95 (20.5)		0.293	1.16 (0.83–1.61)	0.378
	HR (95%CI)	1.18 (0.87–1.61)					
BARC type 3–5 bleeding	no. (%)	78 (7.9)	45 (9.7)		0.07	1.20 (0.76–1.89)	0.445
	HR (95%CI)	1.28 (0.82-2.0)					

*CA-AKI* contrast-associated acute kidney injury, *BARC* Bleeding Academic Research Consortium

*Adjusted hazard risk (HR) for age, hypertension, sex, previous MI, eGFR < 60 mL/min, peripheral artery disease, Killip class ≥ 2, culprit lesion vessel, clinical presentation

### Clinical outcomes according to randomization arm and the diagnosis of diabetes mellitus

**Fig. 1:**
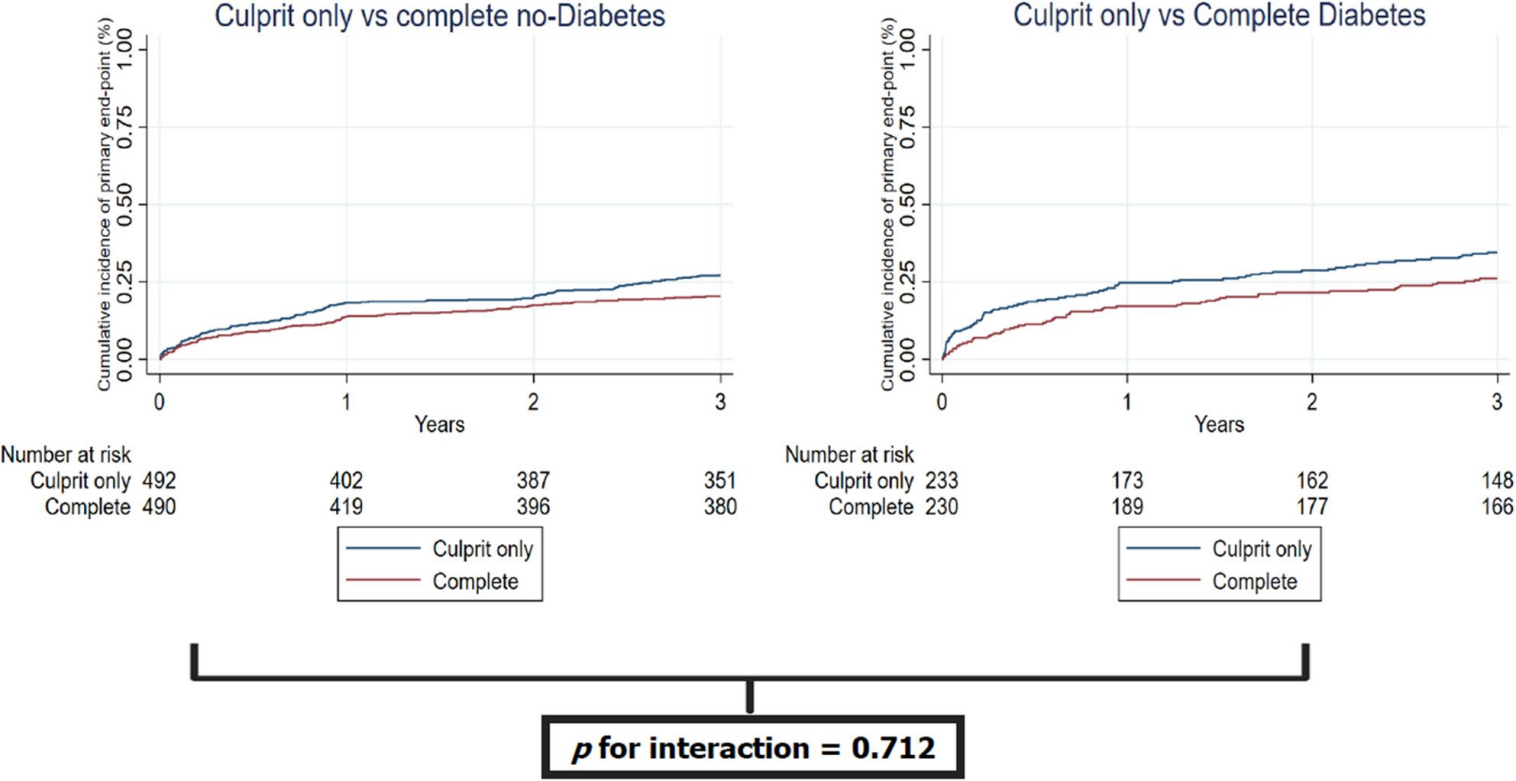
Occurence of primary endpoint according to the presence of diabetes mellitus and randomization arm

**Fig. 2:**
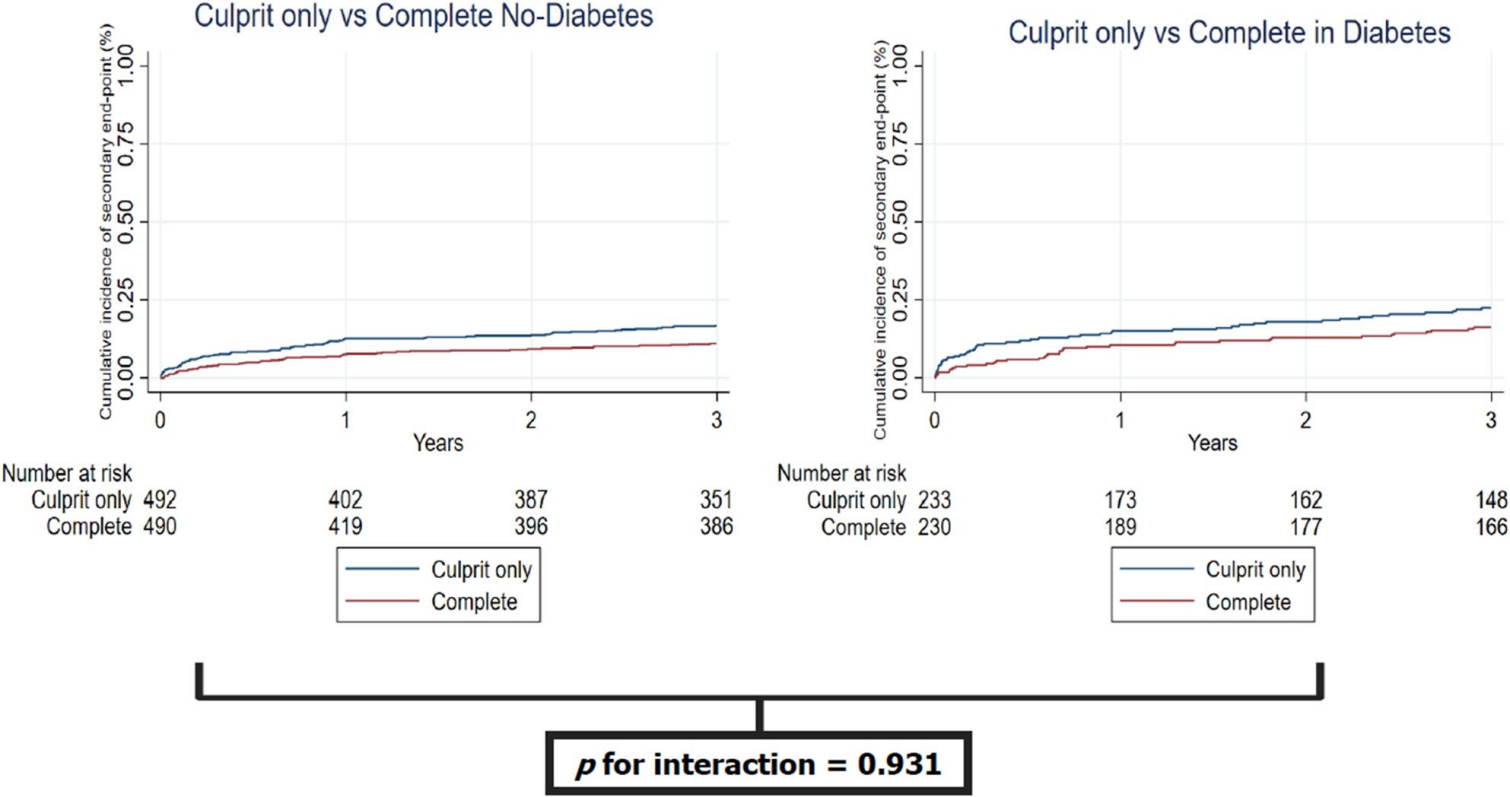
Occurence of key secondary endpoint according to the presence of diabetes mellitus and randomization arm

Complete revascularization reduced the primary endpoint compared to the culprit-only strategy in patients without diabetes (HR 0.75, 95%CI 0.58–0.97), with a similar effect in patients with diabetes (HR 0.70, 95%CI 0.50–0.97) (Table 4; Fig. 1). In absolute terms, physiology-guided complete revascularization reduced the 3-year risk of the primary endpoint from 35.2% to 27.0% in patients with diabetes, corresponding to an absolute risk reduction of 8.2% and a number needed to treat of approximately 12 to prevent one primary endpoint event. There was no heterogeneity of treatment effect on primary endpoint according to the presence of diabetes mellitus (p for interaction 0.712). Same findings were observed for the key secondary endpoint (p for interaction 0.931) (Table 4; Fig. 2). No signal of heterogeneity with respect to the presence of diabetes was observed for the other secondary and safety endpoints (Table 4).

**Table 4:** Clinical outcomes according to the presence of diabetes mellitus and randomization arm

Outcome		No diabetes (*n* = 982)				Diabetes (*n* = 463)						
		Culprit only (*n* = 492)	Complete (*n* = 490)		*p*	Culprit only (*n* = 233)		Complete (*n* = 230)	*p*	Adjusted HR (95%CI) *	*p*	*p* for interaction
Primary outcome												
Composite of death, myocardial infarction, stroke, or ischemia-driven revascularization	no. (%)	134 (27.2%)	103 (21.0%)		0.028	82 (35.2%)		62 (27.0%)	0.044	0.70 [0.50–0.97]	0.035	0.712
	HR (95%CI)	0.75 [0.58–0.97]				0.71 [0.51–0.99]						
Secondary outcomes												
Cardiovascular death, myocardial infarction	no. (%)	81 (16.5%)	54 (11.0%)		0.013	51 (21.9%)		38 (16.5%)	0.136	0.69 [0.44–1.09]	0.108	0.931
	HR (95%CI)	0.68 [0.48–0.97]				0.71 [0.45–1.11]						
Death	no. (%)	92 (18.7%)	67 (13.7%)		0.004	58 (24.9%)		41 (17.8%)	0.034	0.87 [0.61–1.26]	0.470	0.146
	HR (95%CI)	0.60 [0.43–0.85]				0.84 [0.58–1.21]						
Cardiovascular death	no. (%)	51 (10.4%)	33 (6.7%)		0.042	30 (12.9%)		19 (8.3%)	0.109	0.60 [0.33–1.1]	0.1	0.714
	HR (95%CI)	0.64 [0.41–0.98]				0.62 [0.35–1.11]						
Myocardial infarction	no. (%)	35 (7.1%)	23 (4.7%)		0.453	30 (12.9%)		24 (10.4%)	0.852	0.92 [0.45–1.89]	0.830	0.828
	HR (95%CI)	0.80 [0.44–1.44]				0.93 [0.45–1.93]						
Stroke	no. (%)	9 (1.8%)	13 (2.7%)		0.427	5 (2.1%)		5 (2.2%)	0.989	0.91 [0.17–4.88]	0.911	0.634
	HR (95%CI)	1.52 [0.54–4.26]				1.01 [0.25–4.03]						
Ischemia-driven coronary revascularization	no. (%)	42 (8.5%)	25 (5.1%)		0.109	25 (10.7%)		20 (8.7%)	0.321	0.72 [0.35–1.50]	0.382	0.861
	HR (95%CI)	0.65 [0.38–1.10]				0.69 [0.33–1.44]						
Heart failure	no. (%)	85 (17.3%)		60 (12.2%)	0.097	58 (24.9%)	43 (18.7%)		0.155	0.68 [0.42–1.11]	0.120	0.839
	HR (95%CI)	0.74 [0.51–1.06]				0.71 [0.44–1.14]						
Stent thrombosis	no. (%)	4 (0.8%)		5 (1.0%)	0.650	5 (2.1%)	3 (1.3%)		NA	NA	NA	NA
	HR (95%CI)	1.51 [0.25–9.04]				NA						
Safety outcomes												
Composite of CA-AKI, stroke, BARC 3–5	no. (%)	107 (21.7%)		118 (24.1%)	0.106	68 (29.2%)	63 (27.4%)		0.690	1.04 [0.69–1.59]	0.837	0.367
	HR (95%CI)	1.29 [0.95–1.75]				1.09 [0.72–1.64]						
CA-AKI	no. (%)	67 (13.6%)		83 (16.9%)	0.044	49 (21%)	46 (20%)		0.339	1.26 [0.76–2.07]	0.372	0.515
	HR (95%CI)	1.48 [1.01–2.17]				1.28 [0.77–2.18]						
BARC-3-5	no. (%)	43 (8.7%)		35 (7.1%)	0.403	21 (9%)	24 (10.4%)		0.805	0.97 [0.47–1.99]	0.938	0.649
	HR (95%CI)	0.79 [0.46–1.37]				1.09 [0.54–2.21]						

*Adjusted hazard risk (HR) for age, hypertension, sex, previous MI, eGFR < 60 ml/min, peripheral artery disease, Killip class < 2 , culprit lesion vessel, clinical presentation*CA-AKI* contrast-associated acute kidney injury,* BARC* Bleeding Academic Research Consortium

## Discussion

In this prespecified analysis of the FIRE trial, diabetes mellitus identified a subgroup of elderly patients with myocardial infarction and multivessel coronary artery disease at substantially higher risk of adverse cardiovascular events. Despite this increased baseline risk, the relative benefit of physiology-guided complete revascularization was preserved, with no evidence of effect modification by diabetes status. These findings are particularly relevant in the context of cardiovascular diabetology, where multivessel coronary disease in patients with diabetes has long been associated with complex anatomy, diffuse atherosclerosis, and adverse outcomes. Diabetic patients typically exhibit accelerated atherogenesis, increased plaque burden, endothelial dysfunction, and microvascular impairment [8]. These pathophysiological features contribute to higher rates of restenosis, stent thrombosis, and recurrent ischemic events, and have historically driven preference toward surgical revascularization in stable multivessel disease [19]. However, the paradigm established by trials such as FREEDOM largely pertains to younger patients with stable coronary artery disease. Recent evidence from the FAME 3 trial further highlights the role of physiology-guided revascularization strategies in patients with multivessel coronary artery disease [20]. In that trial, Fractional Flow Reserve (FFR)-guided PCI was compared with CABG, and the dedicated diabetes substudy provided important insights into outcomes among patients with diabetes [20, 21]. Although the clinical context differs from the present analysis—where physiology-guided complete revascularization was compared with culprit-only treatment in the setting of acute myocardial infarction—both studies emphasize the importance of physiologic lesion assessment to guide revascularization decisions [20, 21]. Taken together, these findings support the concept that physiologic guidance may help optimize revascularization strategies and promote a more individualized approach in patients with diabetes and complex coronary artery disease. The population enrolled in FIRE differs substantially: elderly individuals presenting with acute myocardial infarction, frequently unsuitable for CABG because of age, comorbidities, or frailty. In such patients, the clinical decision is rarely between PCI and CABG; rather, it concerns how to optimize PCI. Within this context, our findings provide important reassurance. Although diabetic patients had higher absolute event rates, physiology-guided complete revascularization achieved consistent relative risk reductions compared with culprit-only PCI. Given the higher baseline risk, this translates into potentially greater absolute benefit among diabetic patients, challenging the perception that multivessel PCI in elderly diabetics is intrinsically less effective. Equally important is the favorable safety profile observed. In a population prone to stent-related complications and contrast-associated renal injury, physiology-guided complete revascularization did not increase the risk of stent thrombosis, bleeding, stroke, or contrast-associated acute kidney injury. These findings may reflect two complementary mechanisms. First, physiology-guided lesion selection limits treatment to hemodynamically relevant stenoses, reducing unnecessary stent implantation and procedural complexity. In diabetic patients with diffuse coronary disease, this selective approach may be particularly advantageous by avoiding excessive metal burden and minimizing inflammatory and thrombotic stimuli. Second, the use of contemporary sirolimus-eluting stents with bioresorbable polymer (Supraflex Cruz, Sahajanand Medical Technologies Ltd.) likely contributed to the low rates of restenosis and stent thrombosis, even in this high-risk population. Importantly, this contemporary PCI strategy differs markedly from the context of the FREEDOM trial, which established the paradigm favoring CABG in diabetes. FREEDOM enrolled younger patients with stable CAD (median age 63 years) and PCI was performed with first-generation drug-eluting stents without physiologic lesion assessment. Although the individual components of the composite endpoint did not demonstrate large differences within the diabetes subgroup, the direction of effect remained consistent with the overall trial results. Furthermore, no clear difference in cardiovascular mortality was observed between treatment arms, suggesting that the observed mortality signal should be interpreted cautiously and may partly reflect the limited number of events within this subgroup. From a pathophysiological perspective, coronary physiology may be especially valuable in diabetes. Diffuse epicardial disease and microvascular dysfunction can render angiographic assessment misleading, potentially leading to overestimation of lesion severity and excessive revascularization. A physiology-guided strategy helps discriminate truly ischemia-producing lesions from angiographically severe but functionally irrelevant stenoses, thereby improving the appropriateness of revascularization. In elderly diabetic patients, in whom procedural risk is nontrivial, this precision becomes clinically meaningful. Taken together, these results suggest that diabetes should not be viewed as a contraindication to complete percutaneous revascularization in elderly patients with myocardial infarction. When guided by coronary physiology and supported by contemporary stent platforms, multivessel PCI appears both effective and safe in this high-risk group. Rather than prompting therapeutic conservatism, the presence of diabetes may reinforce the importance of physiology-guided decision-making to optimize revascularization strategy.

### Study limitations

Several limitations should be acknowledged. First, this was a prespecified subgroup analysis and, although based on a randomized controlled trial, it was not specifically powered to detect differences in treatment effect within the diabetic subgroup. Consequently, the absence of a statistically significant interaction should be interpreted with caution, and modest heterogeneity cannot be definitively excluded. Second, the trial enrolled elderly patients with myocardial infarction and multivessel disease; therefore, extrapolation to younger diabetic populations or to patients with stable coronary artery disease should be made carefully. Third, while the study demonstrated consistent benefit on composite ischemic endpoints, the number of individual hard events (e.g., death or myocardial infarction) within subgroups was limited, which may restrict precision of estimates. Fourth, although physiology-guided PCI reduced unnecessary revascularization, the trial design does not allow definitive mechanistic conclusions regarding the relative contributions of lesion selection versus stent technology to the observed outcomes. Finally, the FIRE trial compared two percutaneous revascularization strategies (physiology-guided complete revascularization versus culprit-only PCI) and was not designed to evaluate PCI versus CABG. Therefore, the present analysis cannot determine the optimal revascularization modality for patients with diabetes, acute myocardial infarction, and multivessel coronary artery disease. Residual confounding related to diabetes severity, glycemic control, or duration of disease cannot be fully excluded, as detailed metabolic parameters were not the primary focus of the trial.

## Conclusions

In older patients with myocardial infarction and multivessel coronary artery disease, physiology-guided complete revascularization consistently reduced ischemic events irrespective of diabetes status. In this prespecified analysis of the FIRE trial, diabetes mellitus was common and associated with substantially higher ischemic risk; however, the relative benefit of complete revascularization was preserved, with no signal of excess procedural or safety events. These findings reinforce the main results of the FIRE trial and suggest that diabetes should not discourage the use of physiology-guided complete revascularization in elderly patients with myocardial infarction and multivessel disease.

## Data Availability

The data underlying this article are available on reasonable request to the FIRE study Executive Committee.

## Abbreviations

MI: Myocardial infarction
FIRE: Functional assessment in elderly mi patients with multivessel disease
RCT: Randomized clinical trial
PCI: Percutaneous coronary intervention
CAD: Coronary artery disease
ARC: Academic research consortium
BARC: Bleeding academic research consortium
CA-AKI: Contrast-associated acute kidney injury
CABG: Coronary artery bypass grafting
ACS: Acute coronary syndrome
STEMI: ST-segment elevation myocardial infarction
NSTEMI: Non-ST-segment elevation myocardial infarction
eGFR: Estimated glomerular filtration rate
HR: Hazard ratio
CI: Confidence interval
FFR: Fractional flow reserve
